# Comment on Myocardial Perfusion Study in Obese Patients without Known Cardiac Ischemia

**DOI:** 10.5935/abc.20190082

**Published:** 2019-05

**Authors:** Claudio Tinoco Mesquita, Gustavo Gavina da Cruz

**Affiliations:** 1 Universidade Federal Fluminense - Departamento de Radiologia, Niterói, RJ - Brazil; 2 Hospital Pró-cardíaco, Rio de Janeiro, RJ - Brazil; 3 Universidade Federal Fluminense - Pós-Graduação em Ciências Cardiovasculares, Niterói, RJ - Brazil; 4 Fundação Técnico Educacional Souza Marques, Rio de Janeiro, RJ - Brazil

**Keywords:** Coronary Artery Disease, Myocardial Perfusion Imaging, Obesity/mortality, Myocardial Ischemia, Diabetes Mellitus

*"All human knowledge is fallible and therefore uncertain. It follows that we must distinguish sharply between truth and certainty... This is the task of scientific activity. Hence, we can say: our aim as scientists is objective truth; more truth, more interesting truth, more intelligible truth. We cannot reasonably aim at certainty*."

Karl Popper

We congratulate Dippe et al.^1^ for their work that addressed the role of myocardial scintigraphy in the diagnosis of myocardial ischemia in obese patients.^1^ Despite limitations, body mass index (BMI) has been the most used anthropometric tool for assessing nutritional status in adults.^2^ Epidemiologic studies have identified high BMI as a risk factor for an expanding set of chronic diseases, including cardiovascular disease and diabetes mellitus. The Global Burden of Disease (GBD) Obesity Collaborators found that excess body weight accounted for about 4 million deaths in 2015. Nearly 70% of these deaths were due to cardiovascular disease, and more than 60% of them occurred among obese persons (BMI ≥ 30 Kg/m^2^).^3^ The use of a database of consecutive patients provides a sample of obese patients from the real-world scenario and portrays the current clinical practice in which the cardiologist faces major diagnostic challenges in obese patients. All diagnostic methods have significant challenges in obese patients such as the limitation of the acoustic window in the echocardiogram, higher incidence of photon attenuation on computed tomography and myocardial scintigraphy and bore size limitations to cardiac resonance imaging. Radiation-sparing techniques are more difficult to use in heavier patients.^4^ The finding that clinical data such as the presence of diabetes mellitus, older age and typical symptoms of angina highlights the need of careful clinical evaluation in order to adequate request ischemic screening tests in patients with suspected coronary artery disease, especially in the obese. Another important finding of their study was the absence of association between obesity alone, especially in the group with BMI greater than 40, with the presence of ischemia. A technical aspect that was not clear in the article and whether the authors used the prone acquisition when there was doubt about the presence of breast attenuation and also the technique used to quantify the visual or automatic ischemia.

In an editorial about this article, Hueb^5^ points out the multiple mechanisms involved in the pathophysiology of myocardial ischemia, including the microvascular mechanisms that determine ischemia in patients with epicardial coronary arteries without obstruction. Functional methods are important in the identification of microvascular ischemic abnormalities, which have diagnostic and prognostic value, especially in diabetic patients and in patients with multiple risk factors. Functional imaging is superior to anatomic imaging in patients with microvascular disease because of their focus on different levels of the ischemic cascade including wall motion abnormalities (echocardiography and stress cardiac magnetic resonance), relative perfusion abnormalities (stress cardiac magnetic resonance and single-photon emission computed tomography), and changes in physiological absolute regional myocardial perfusion (PET).^6^ The creation of the patient-centered imaging culture that prioritizes patient safety and effectiveness requires the understanding of the better diagnostic techniques for every clinical need.^7^

Karl Popper stated that science is composed of transient truths. The role of scientists is to prove the falsifiability of their findings and others in the search of a more intelligible true. In the absence of contrary evidence, current evidence points that invasive treatment in patients with myocardial ischemia area greater than 10% is associated with better prognosis in comparison with medical management alone. The results of the ISCHEMIA study to be published in the near future should provide additional new scientific evidence regarding whether an invasive management strategy improves clinical outcomes when added to optimal medical therapy in patients moderate or severe ischemia.^8^
