# Supplementary material for: Comment on Myocardial Perfusion Study in Obese Patients without Known Cardiac Ischemia
Source: Arq Bras Cardiol. 2019 May;112(5):597–9. doi: 10.5935/abc.20190082 (PMC6555579; doi:10.5935/abc.20190082)
Supplement: Related texts

# Reply

**Authors:** Tufi Dippe Junior, João Vicente Vítola

Regarding our manuscript,^1^ we would like to make some comments about the letter sent to the editor by Universidade Federal Fluminense (UFF) and about the short editorial written by Dr. Whady Rueb.^2^

Although body mass index (BMI) correlates with the percentage of body fat in most individuals, the limitations of this index is widely known.^3-5^ On the other hand, major cohort, prospective and observational studies, such as the Framingham study^6^ and the Nurse's Health Study,^7^ used BMI as a diagnostic parameter for obesity, demonstrating a nearly linear relationship between BMI and coronary artery disease (CAD) from a value equal to or greater than 25 kg/m^2^.

The World Health Organization (WHO) uses BMI for the diagnosis and classification of obesity.^8^ In our study,^1^ which evaluated 5,526 obese patients undergoing myocardial perfusion scintigraphy, one of the largest samples ever published in the world literature, 29.7% of the individuals had BMI equal to or greater than 35 kg/m^2^.

Regarding the questioning of the UFF colleagues, we pointed out that the limitations of the manuscript include that our patients were not submitted to attenuation correction techniques routinely.

Before we make any specific comments on the short editorial, we would like to emphasize our deep admiration for Dr. Whady Hueb, a Brazilian scientist of great importance for the world cardiology, who we highly appreciate and respect. We would also like to emphasize, with no reservations, his contribution to the international literature with the MASS study,^9^ quoted and admired all over the world. Today, among other things, the MASS study allows us to work together on the ISCHEMIA study,^10^ on which both Dr. Whady Hueb's and our group worked hard for a successful completion.

Regarding the minieditorial on our study, we would first like to make some comments about the tests recommended and the perfusional abnormality rate we found.

Note that our registry in Curitiba, which is certainly one of the largest nuclear cardiology registries in the world, includes patients referred to our diagnostic center, about whom we have no control over which are the tests to be recommended, as this is the responsibility of the referring clinician. (I do not understand this)

Besides that, we cannot infer that the tests have been inappropriately recommended based on 77% of normal scintigraphies. We are sure that this data should not be used as a criticism of our study, since in many clinical situations this is exactly the information sought by the clinician requesting a provocative ischemia test, that is, the absence of ischemia can avoid unnecessary anatomical evaluations, such as cineangiocorography, for example.

It is true that many of these patients with suspected CAD could have their disease ruled out by coronary angiography. Unfortunately, this practice is still limited in our country, because of the restrictions imposed by health insurance plans or unavailability in the public health system (SUS). We believe that this would be an excellent way to "rule out" CAD, avoiding additional tests, including myocardial perfusion scintigraphy itself.

Although our perfusion abnormality rate (23%) was considered low by Dr. Whady Hueb, it is nearly three times greater than that found in reference laboratories in the United States, as found by the Cedars Sinai Hospital registry, which revealed about 8.7% of perfusion abnormalities.^11^ Similarly, the randomized study PROMISE^12^ found a perfusion abnormality rate close to 10% in symptomatic patients.

In our sample, 31% of the patients were known diabetics, and this certainly differentiates our group from other studies, and helps us understand our high abnormality rate.

Another excerpt of the short editorial reads: *based on this data, by applying a "creative statistics", they found a 245% risk increase for typical angina*.

Note that nowhere in the manuscript we mentioned that a perfusional abnormality would increase the risk of typical angina. We have published that the patients who reported typical angina before the test, compared to asymptomatic patients (reference) had 245% higher chances of having abnormal myocardial perfusion (odds ratio of 2.45 [1.82-3.31], see page 125 of the manuscript, table 4).^1^ There is no "creative statistics" at all. This conclusion was reached after multivariate logistic regression analysis. It is pure statistics.

Finally, we would like to thank UFF for the letter sent to the editor of Arquivos Brasileiros de Cardiologia and to Dr. Whady Hueb for his short editorial. The productive discussion and scientific production certainly help to add further value to our admired Brazilian cardiology.

## References

[1] Dippe Jr T, Leinig C, Cerci RJ, Lafitte AS Jr, Vítola JV (2019). Estudo de perfusão miocárdica em obesos sem
doença cardíaca isquêmica conhecida. Arq Bras Cardiol.

[2] Hueb W (2019). A cintilografia de perfusão miocárdica com
tomografia computadorizada por fóton unico. Ferramenta
diagnóstica antecipando a doença. Arq Bras Cardiol.

[3] Gallagher D, Heymsfield SB, Heo M, Jebb SA, Murgatroyd PR, Sakamoto Y (2000). Healthy percentage body fat ranges: an approach for
Developing guidelines based on body mass index. Am J Clin Nutr.

[4] Shiwaku K, Kitajima K, Yamane Y (2004). Appropriate BMI for Asian populations. Lancet.

[5] Deurenberg P, Yap M, Wang J, Lin FP, Schmidt G (1999). The impact of body build on the relationship between body
mass index and percent body fat. Int J Obes Relat Metab Disord.

[6] Hubert HB, Feinleib M, McNamara PM, Castelli WP (1983). The obesity an independent risk factor for cardiovascular
disease: a 26-year follow-up of participants in the Framingham Heart
Study. Circulation.

[7] Manson JE, Colditz GA, Stampfer MJ, Willett WC, Rosner B, Monson RR (1990). A prospective study of obesity and risk of coronary heart
disease in women. N Engl J Med.

[8] World Health Organization. (WHO) (2000). Obesity: Preventing and managing the global epidemic. Report of the
World Health Organization Consultation.

[9] Hueb W, Bellotti G, de Oliveira SA, Ariê S, Albuquerque CP, Jatene AD, Pileggi F (1995). The Medicine, Angioplasty or Surgery Study (MASS): a
prospective, randomized trial of medical therapy, balloon angioplasty or
bypass surgery for single proximal left anterior descending artery
stenoses. J Am Coll Cardiol.

[10] Hochman JS, Harmony RS, Bangalore S, O'Brien SM, Alexander KP, Senior R (2019). Baseline Characteristics and risk profiles of participants in
the ISCHEMIA Randomized Clinical Trial. JAMA Cardiol.

[11] Rozanski A, Gransar H, Hayes SW, Min J, Friedman JD, Thomson LF (2013). Temporal trends in the frequency of inducible myocardial
ischemia during cardiac stress testing: 1991 to 2009. J Am Coll Cardiol.

[12] Douglas PS, Hoffmann U, Patel MR, Mank DB, Al-Khalidi HR, Cavanaugh B, PROMISE Investigators (2015). Outcomes of anatomical versus functional testing for coronary
artery disease. N Engl J Med.
